# Plasma Neutrophil Gelatinase-Associated Lipocalin Associates with New-Onset Chronic Kidney Disease in the General Population

**DOI:** 10.3390/biom13020338

**Published:** 2023-02-09

**Authors:** Arno R. Bourgonje, Amaal E. Abdulle, Martin F. Bourgonje, Lyanne M. Kieneker, Sacha la Bastide-van Gemert, Sanne J. Gordijn, Clara Hidden, Tom Nilsen, Ron T. Gansevoort, Douwe J. Mulder, Robin P. F. Dullaart, Martin H. de Borst, Stephan J. L. Bakker, Harry van Goor

**Affiliations:** 1 Department of Gastroenterology and Hepatology, University of Groningen, University Medical Center Groningen, 9713 GZ Groningen, The Netherlands; 2 Department of Internal Medicine, Division of Vascular Medicine, University of Groningen, University Medical Center Groningen, 9713 GZ Groningen, The Netherlands; 3 Department of Pathology and Medical Biology, University of Groningen, University Medical Center Groningen, 9713 GZ Groningen, The Netherlands; 4 Department of Internal Medicine, Division of Nephrology, University of Groningen, University Medical Center Groningen, 9713 GZ Groningen, The Netherlands; 5 Department of Epidemiology, University of Groningen, University Medical Center Groningen, 9713 GZ Groningen, The Netherlands; 6 Department of Gynecology and Obstetrics, University of Groningen, University Medical Center Groningen, 9713 GZ Groningen, The Netherlands; 7 Gentian AS, 1596 Moss, Norway; 8 Department of Internal Medicine, Division of Endocrinology, University of Groningen, University Medical Center Groningen, 9713 GZ Groningen, The Netherlands

**Keywords:** chronic kidney disease, neutrophil gelatinase-associated lipocalin, epidemiology, NGAL, CKD

## Abstract

Circulating levels of neutrophil gelatinase-associated lipocalin (NGAL) have been associated with acute kidney injury and the severity and progression of chronic kidney disease (CKD). This study investigated its potential utility as a biomarker for the risk of new-onset CKD in a population-based cohort study. Individuals without CKD at baseline (n = 4660) who participated in the Prevention of REnal and Vascular ENd-stage Disease (PREVEND) prospective population-based cohort study in the Netherlands were included. Baseline plasma NGAL concentrations were investigated for their associations with new-onset CKD, defined as a composite outcome of an estimated glomerular filtration rate (eGFR) < 60 mL/min/1.73 m^2^, urinary albumin excretion (UAE) > 30 mg/24-h, or both. Mean (±SD) plasma NGAL concentrations were 104.0 (±34.7) μg/L and median eGFR was 96 [IQR: 85.3–105.8] mL/min/1.73 m^2^. After median follow-up of 8.3 [IQR: 7.8–8.9] years, 467 participants developed new-onset CKD. Plasma NGAL concentrations were significantly associated with an increased risk of new-onset CKD (hazard ratio [HR] per doubling 1.35 [95% CI: 1.11–1.63], *p* = 0.002), even after adjustment for potentially confounding factors (1.37 [1.09–1.73], *p* = 0.007) except baseline eGFR (1.09 [0.86–1.37], *p* = 0.490). In secondary analyses, plasma NGAL concentrations were significantly associated with new-onset CKD as defined by eGFR < 60 mL/min/1.73 m^2^ alone (adjusted HR per doubling 2.54 [1.69–3.80], *p* < 0.001), which was abrogated after adjustment for eGFR (1.05 [0.69–1.59], *p* = 0.828), also when UAE > 30 mg/24-h was set as individual outcome (1.05 [0.82–1.35], *p* = 0.705). Higher plasma NGAL concentrations are associated with an increased risk of developing CKD in the general population. This association is dependent on renal function, and mainly driven by new-onset CKD as defined by renal function decline.

## 1. Introduction

Chronic kidney disease (CKD) is a debilitating disease affecting almost one billion individuals globally [1]. It is accompanied by a high degree of cardiovascular morbidity and is associated with premature mortality, emphasizing the importance of early identification and staging of individuals affected by CKD. Since the etiology of CKD is of multifaceted nature and remains incompletely understood, there is an urgent need for biomarkers to identify at-risk individuals for CKD in the general population in early stages [2]. Identification of such individuals in pre-diagnostic phases would allow timely treatment initiation and the implementation of preventive measures to delay or prevent the necessity of dialysis or transplantation [3].

Neutrophil gelatinase-associated lipocalin (NGAL, also known as lipocalin-2) is a granular glycoprotein produced in the bone marrow during myelopoiesis, stored by neutrophils and renal tubular cells, and released in the systemic circulation in response to tubular injury [4]. NGAL in blood and urine is a well-established biomarker of acute kidney injury (AKI), both for short-term and persistent AKI, and across various patient populations [5,6,7,8]. Previously, blood and urinary NGAL levels have been described as markers for delayed graft function, graft failure, and mortality in kidney transplant recipients [9]. In addition, NGAL levels have been associated with the severity and progression of CKD [10,11,12,13], suggesting that NGAL may also reflect ongoing renal damage underlying CKD progression. Importantly, elevated circulating and urinary NGAL levels have been observed in the absence of overt signs of CKD (e.g., increased serum creatinine levels or decreased estimated glomerular filtration rate [eGFR]), rendering it a potentially attractive biomarker for pre-diagnostic CKD [10,13]. For example, it has been hypothesized that increased NGAL levels are the result of sustained production by injured renal tubular cells, whereas renal function impairment would then passively result from a general loss of functional cells or nephrons [14,15]. Previously, a nested case–control study evaluated the potential utility of urinary NGAL as biomarker for incident CKD stage III [16], showing a significant association between higher NGAL levels and risk of incident CKD. This study, however, was limited by sample size and did not take into account potential loss-to-follow-up due to its case–control design. Until now, the potential utility of circulating NGAL levels in relation to new-onset CKD after long-term follow-up has remained unexplored.

Therefore, we hypothesized that circulating levels of NGAL may have merit as a prognostic biomarker for long-term renal health. Aside from reflecting tubular injury, NGAL may also reflect chronic low-grade inflammation, which could underlie the development of noncommunicable diseases such as CKD. In the current study, we aimed to investigate the association between plasma NGAL levels and the risk of new-onset CKD in individuals derived from the general population. 

## 2. Materials and Methods

### 2.1. Study Design and Population

This study was performed as part of the Prevention of REnal and Vascular ENd-stage Disease (PREVEND) cohort study, which is a large-scale, prospective population-based cohort study that commenced in 1997 in Groningen, the Netherlands [17]. The PREVEND study was primarily aimed to study the utility of urinary albumin excretion as indicator of the future occurrence of renal and cardiovascular diseases. The PREVEND study comprises data on a wealth of parameters that are relevant to cardiorenal diseases. In the period from 1997–1998, 85,421 inhabitants of the city of Groningen aged 28–75 years were contacted and requested to complete a short questionnaire asking information on demographics and history of cardiovascular diseases, as well as to send in a first morning void sample. A total of 40,856 individuals (47.8%) responded, from which 7786 individuals with urinary albumin concentrations (UAC) > 10 mg/L and a randomly selected control group of 3395 individuals with UAC < 10 mg/L were invited to participate in subsequent study screening investigations at the outpatient research clinic of the University Medical Center Groningen (UMCG). This screening program was completed by 8592 participants (n = 6000 with UAC > 10 mg/L and n = 2592 with UAC < 10 mg/L), which together formed the total study cohort. In the period of 2001–2003, a second round of study investigations was organized aiming to collect additional data and biomaterials (e.g., blood, urine) from 6894 of these participants. This second study round served as the baseline for the present study. From this cohort of participants, patients with established CKD defined as having an estimated glomerular filtration rate (eGFR) < 60 mL/min/1.73 m^2^ (n = 1082) or unknown CKD status (n = 337) were excluded. Furthermore, participants in whom plasma NGAL levels could not be determined (n = 815) due to either missing samples or insufficient sample volumes) were excluded. This resulted in a final sample size of n = 4660 participants for analysis with a study follow-up that ended on 1 January 2011. The PREVEND study was approved by the Institutional Review Board of the UMCG (full name in Dutch: “Medisch Ethische Toetsingscommissie”, abbreviated as “METc”, IRB no. 01/139). All participants provided written informed consent for study participation. The study was conducted according to the principles of the Declaration of Helsinki. The study reporting was in accordance with the EQUATOR guideline: the Strengthening the Reporting of Observational Studies in Epidemiology (STROBE) [18].

### 2.2. Data Collection

Demographic-, medical- (e.g., history of cardiorenal diseases and diabetes), lifestyle-related (e.g., smoking behavior) and anthropometric characteristics (e.g., body height, weight and waist circumference) were collected using questionnaires that participants completed at baseline. Blood pressure was measured in supine position for 8 min using the Dinamap XL Model 9300 series device (Johnson & Johnson Medical, Tampa, FL, USA). Smoking behavior was denoted as “never”, “former”, or “current”. Waist circumference was measured on the bare skin at the natural indentation between the 10th rib and the iliac crest. Blood pressure was determined every minute, from which the average of the last two measurements was taken for analyses, as previously described [19,20]. Medication use was principally self-reported but was combined with information from a pharmacy-dispensing registry that had complete information available on drug usage of >95% of participants of the PREVEND study [21,22]. Fasting venous plasma and serum samples were collected from study participants, centrifuged at 4 °C, and stored at −80 °C until further analysis, while urine samples were stored at −20 °C until analysis. Urinary albumin excretion (UAE) was measured by nephelometry (Dade Behring Diagnostics, Marburg, Germany). UAE was measured twice using two consecutive 24 h urine collections, from which the average was taken for statistical analyses. Serum creatinine levels were measured using an enzymatic method (Roche Modular, Roche Diagnostics, Mannheim, Germany). Serum cystatin C levels were measured on a modular analyzer (Roche Diagnostics) using the Gentian Cystatin C Immunoassay (Gentian AS, Moss, Norway). Calibration standards for cystatin C were used following manufacturer’s instructions and according to the International Federation of Clinical Chemistry Working Group for Standardization of Serum Cystatin C [23]. High-sensitive C-reactive protein (hs-CRP) levels were determined by nephelometry (Dade Behring Diagnostics, Marburg, Germany).

### 2.3. Measurements of NGAL

Plasma concentrations of NGAL were measured with a turbidimetric immunoassay leveraging a Mindray BS-400 analyzer (Gentian AS, Moss, Norway). This NGAL assay has been calibrated to the commercially available Bioporto NGAL test using a previously described value transfer protocol [24]. The NGAL calibrator, in turn, has been validated based on the Clinical and Laboratory Standards Institute guidelines in conjunction with external validation performed by individual laboratories [25]. The lower limit of detection (LLoD) of NGAL was 6.3 ng/mL and intra-assay and inter-assay coefficients of variation (CV) were between 0.4–5.2% and 0.6–7.1%, respectively.

### 2.4. Study Outcomes and Definitions

New-onset CKD served as primary outcome and was defined as the first occurrence of either an estimated glomerular filtration rate (eGFR) < 60 mL/min/1.73 m^2^, UAE > 30 mg/24-h, or both. The first occurrence of an eGFR < 60 mL/min/1.73 m^2^ or UAE > 30 mg/24-h individually served as secondary outcomes. Here, the eGFR was calculated using the combined cystatin C-based Chronic Kidney Disease Epidemiology Collaboration (CKD-EPI) equation [26]. Type 2 diabetes mellitus (T2DM) was defined either as a fasting glucose concentration ≥ 7.0 mmol/L, a non-fasting glucose level of ≥11.1 mmol/L, or the use of antidiabetic drugs based on pharmacy- or self-reports according to the American Diabetes Association (ADA) guidelines [27]. Hypercholesterolemia was defined either as serum total cholesterol levels ≥ 6.5 mmol/L, serum high-density lipoprotein (HDL)-cholesterol levels ≤ 0.9 mmol/L or the use of lipid-lowering drugs.

### 2.5. Statistical Analyses

Baseline demographic, anthropometric, medical, and laboratory characteristics of study participants were presented as mean ± standard deviations (SD), medians with interquartile ranges (IQR) in case of skewed variables or as proportions with corresponding percentages (%). Normality assessment was performed by visual inspection of normal probability (Q-Q) plots and histograms. Differences in baseline characteristics across tertiles of plasma NGAL concentrations were tested using one-way analysis of variance (ANOVA), Kruskal–Wallis tests, and chi-square tests, where appropriate. Survival distributions for participants across tertiles of plasma NGAL concentrations were evaluated using Kaplan–Meier survival curves, which were compared with tertiles using log-rank tests. Survival time was defined as from baseline (time of plasma sample collection) until the date of the last examination that participants attended, at the incidence of CKD, death, or 1 January 2011 (end of follow-up). Plasma NGAL concentrations were 2log-transformed before entry into subsequent analyses to facilitate results interpretation (expressed as per doubling in NGAL concentration). Cox proportional hazards regression analyses were performed to test associations between plasma NGAL and the risk of new-onset CKD (as composite outcome, and by individual outcomes based on either eGFR or UAE), where results were expressed as hazard ratios (HRs) (per doubling) with corresponding 95% confidence intervals (CIs). For each predictor, the proportionality of hazards assumption was checked to confirm absence of violation. Stratified analyses were subsequently performed to test associations across relevant subgroups and explore potential interactions by fitting models containing interaction terms (where *p*_interaction_ < 0.05 was considered indicative of significant effect-modification). Restricted cubic splines (RCS) with three knots were additionally fitted to evaluate potential nonlinearity of the observed associations in Cox proportional hazards regression models. Nonlinearity was statistically assessed using likelihood ratio tests, in which nested models were compared with each other using linear or linear and cubic spline terms. Data analysis and data visualization was performed using SPSS Statistics 28.0 software package (SPSS Inc., Chicago, IL, USA), R (v.4.0.1, Vienna, Austria) and the Python programming language (v.3.8.6, Python Software Foundation, https://www.python.org, accessed on 9 January 2023), using the *pandas* (v.0.12.3), *numpy* (v.1.20.0), *matplotlib* (v.3.4.1), *seaborn* (v.0.11.1) and *zepid* (v.0.9.0) packages. Two-tailed *p*-values ≤ 0.05 were considered statistically significant. 

### 2.6. Selection of Potentially Confounding Variables: Directed Acyclic Graph (DAG)

**Figure 1:**
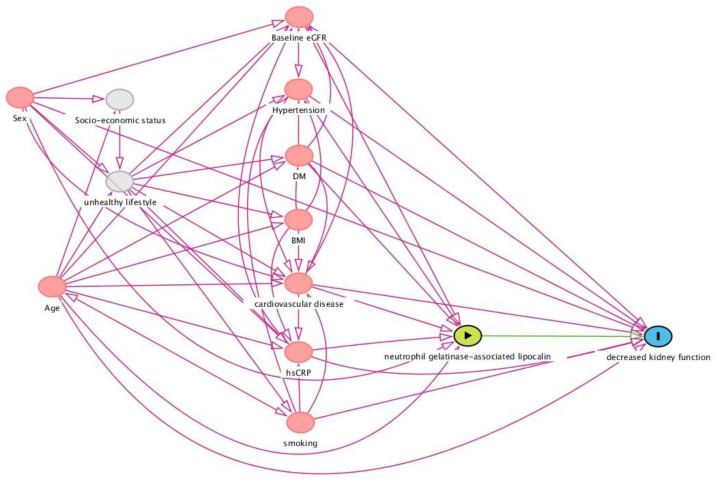
Direct acyclic graph (DAG) demonstrating the hypothesized causal relationships underlying the studied association between circulating levels of NGAL and the risk of new-onset CKD in the general population. Based on this framework, a distinct set of confounding variables was identified and subsequently conditioned for in statistical analysis (see text). Abbreviations: BMI, body mass index; CKD, chronic kidney disease; DM, diabetes mellitus; hs-CRP; high-sensitive C-reactive protein; NGAL, neutrophil gelatinase-associated lipocalin.

A directed acyclic graph (DAG) was constructed to identify potentially confounding variables warranting consideration while estimating the study’s outcome of interest: new-onset CKD [28]. DAGs are causal models and serve as theoretical basis by pre-defining the involved causal mechanisms hypothesized to underlie the variables of interest (Figure 1). In the DAG, arrows depict the hypothesized causal (direct) effects between variables, whereas the absence of such arrows represent the assumption of no such direct effect. In this study, we aimed to investigate the association between plasma NGAL levels and new-onset CKD, for which a distinct set of potentially confounding variables was identified and conditioned for in order to approach an unconfounded effect estimate in statistical analyses [29,30,31,32,33,34,35]. Based on this DAG framework, we selected the following variables as covariates in the analysis, in addition to age and sex: a history of cardiovascular disease, history of diabetes, the presence of hypertension, hs-CRP reflecting systemic inflammation, and baseline eGFR reflecting renal function.

## 3. Results

### 3.1. Baseline Study Population Characteristics

Baseline characteristics of the study population divided by tertiles of plasma NGAL concentrations (T1: <87.6 μg/L; T2: 87.6–112.6 μg/L; T3: >112.6 μg/L) are presented in Table 1. Mean (±SD) plasma NGAL concentrations were 104.0 (±34.7) μg/L (median: 99.3 μg/L; full range: 0–402.8 μg/L). Median age of participants was 50.0 [IQR: 42.2–58.8] years and 2513 (53.9%) participants were female. Median eGFR was 96 [IQR: 85.3–105.8] mL/min/1.73 m^2^, and eGFR was lowest in participants within the highest (T3) tertile of plasma NGAL concentrations (*p* < 0.001) compared to participants within T1 or T2. Median UAE was 7.7 [5.8–11.2] mg/L, which did not significantly differ among plasma NGAL tertiles (*p* = 0.409). Participants within the highest (T3) tertile of plasma NGAL concentrations demonstrated the highest rate of new-onset CKD (composite outcome: *p* = 0.029; based on eGFR < 60 mL/min/1.73 m^2^: *p* = 0.002), albeit not when solely defined by UAE > 30 mg/24-h (*p* = 0.483).

### 3.2. Plasma NGAL Concentrations and New-Onset CKD

**Figure 2:**
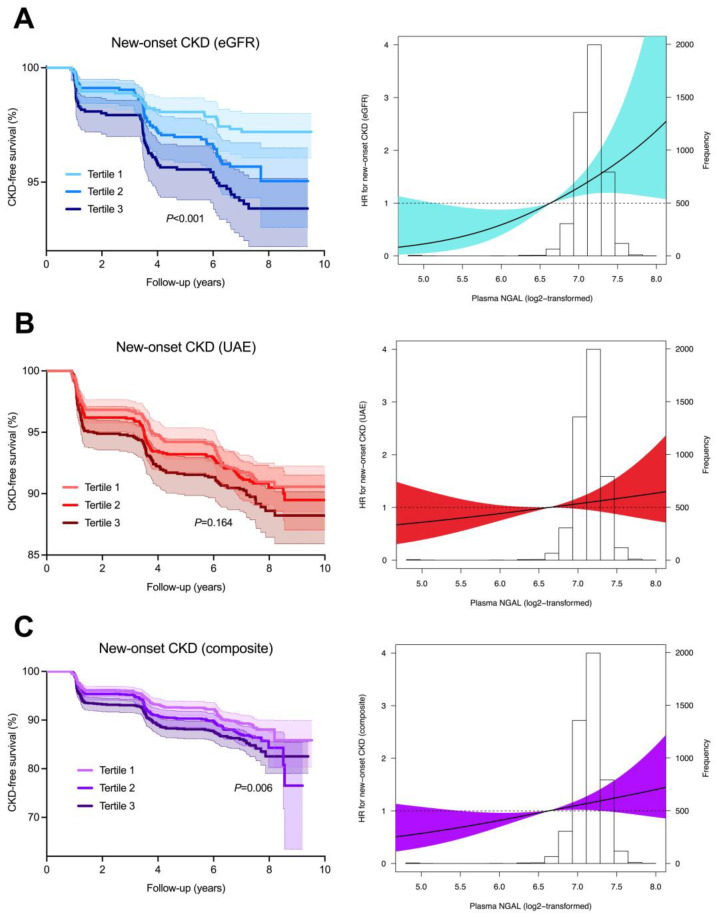
Kaplan-Meier survival curves and restricted cubic splines for the association between plasma NGAL concentrations and the risk of new-onset CKD based on the individual determinants of (**A**) eGFR (<60 mL/min/1.73 m^2^) and (**B**) UAE (>30 mg/24-h) and (**C**) on the composite outcome (eGFR, UAE or both) The highest rates of new-onset CKD were observed in the highest tertiles (T3) of plasma NGAL concentrations (left panels). Restricted cubic splines (RCS) were fitted to test for potential nonlinearity of the association between plasma NGAL concentrations and the risk of new-onset CKD (right panels). Estimated associations were derived from the Cox proportional hazards regression models and RCS with three knots (set at the 0.5th, 50th, and 99.5th percentiles). Likelihood ratio tests for non-linearity were non-significant for all three models (composite: χ^2^ = 0.19, *p* = 0.663; eGFR: χ^2^ = 0.21, *p* = 0.645; UAE: χ^2^ = 0.03, *p* = 0.861). Shaded areas of the RCS curves represent the 95% confidence intervals. Abbreviations: CKD, chronic kidney disease; eGFR, estimated glomerular filtration rate; NGAL, neutrophil gelatinase-associated lipocalin; UAE, urinary albumin excretion.

Over a median follow-up of 8.3 [7.8–8.9] years, a total of 467 participants developed new-onset CKD, among which 151 based on eGFR (<60 mL/min/1.73 m^2^), 349 based on UAE (30 mg/24-h), and 33 based on both. The highest rate of new-onset CKD occurred in the highest tertile of plasma NGAL concentrations (n = 174, 11.4%, *p* < 0.05). Kaplan-Meier survival curves demonstrated statistically significant differences in survival distribution for tertiles of plasma NGAL concentrations and the risk of new-onset CKD, for the composite outcome (*p* = 0.006, log-rank test) and for new-onset CKD defined solely by eGFR (*p* < 0.001, log-rank), but not for new-onset CKD defined by UAE (*p* = 0.164, log-rank) (Figure 2). Cox proportional hazards regression analyses revealed a statistically significant association between plasma NGAL and the risk of new-onset CKD (Table 2A, Model 1, hazard ratio [HR] per doubling of NGAL concentration 1.35 [95% CI: 1.11–1.63], *p* = 0.002). When adjusting for confounding factors (age, sex, history of cardiovascular disease, history of diabetes, presence of hypertension, and hs-CRP), this association remained significant (Table 2A, Model 4, HR per doubling 1.37 [1.09–1.73], *p* = 0.007). However, after additional adjustment for baseline eGFR, this statistically significant association was abrogated (Model 5, HR per doubling 1.09 [0.86–1.37], *p* = 0.490). When these analyses were performed for the individual components of the composite outcome (eGFR < 60 mL/min/1.73 m^2^ and UAE > 30 mg/24-h), a considerably stronger association was found for eGFR as outcome (Table 2B, Model 1, HR per doubling 2.07 [1.47–2.91], *p* < 0.001), whereas for UAE as outcome the association was not statistically significant (Table 2C, Model 1, HR per doubling 1.21 [0.98–1.50], *p* = 0.080). After adjusting for confounding variables, the association between plasma NGAL and new-onset CKD as defined by eGFR < 60 mL/min/1.73 m^2^ remained strong and robust (Table 2B, Model 4, HR per doubling 2.54 [1.69–3.80], *p* < 0.001), but again lost significance after additional adjustment for baseline eGFR (Model 5, 1.05 [0.69–1.59], *p* = 0.828). The association between plasma NGAL and UAE as individual outcome was non-significant after adjustment for confounding factors (Table 2C, Model 4, HR per doubling 1.05 [0.82–1.35], *p* = 0.705), which remained unchanged after additional adjustment for baseline eGFR (Model 5, 1.07 [0.82–1.40], *p* = 0.604). When tertiles of plasma NGAL levels were used instead of plasma NGAL as continuous variable, the highest tertile (T3) of plasma NGAL was consistently significantly associated with the risk of new-onset CKD (as composite outcome and individually by eGFR < 60 mL/min/1.73 m^2^) after adjustment for confounding variables (Table 2A,B, Model 4, HR per doubling 1.51 [1.16–1.96], *p* = 0.002 (composite) and 2.55 [1.60–4.06], *p* < 0.001 (eGFR)), but again vanished after additional adjustment for baseline eGFR (all *p* > 0.05). For all three sets of models with different outcomes of new-onset CKD, no significant deviations from linear associations with the risk of new-onset CKD were observed when restricted cubic splines (RCS) were fitted (composite: *p* = 0.663; eGFR: *p* = 0.645; UAE: *p* = 0.861). 

### 3.3. Stratified Analyses

**Figure 3:**
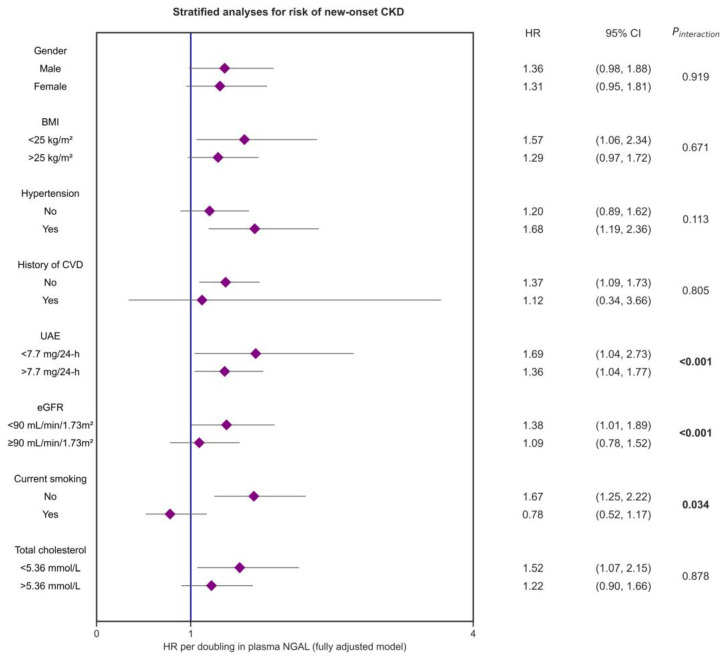
Forest plot showing stratified analyses for the association between plasma NGAL concentrations and the risk of new-onset CKD (composite outcome) across various subgroups. The figure demonstrates hazard ratios (HRs, purple dots) with corresponding 95% confidence intervals (lines). Almost all subgroups showed positive associations between plasma NGAL and the risk of new-onset CKD, except for smokers (albeit nonsignificant). All models were adjusted for selected confounding variables (corresponding with Model 4), including age, sex, a history of cardiovascular disease, history of diabetes, presence of hypertension, and high-sensitive C-reactive protein. Bold *p*-values indicate statistically significant interaction. Abbreviations: BMI, body mass index; CI, confidence interval; CVD, cardiovascular disease; eGFR, estimated glomerular filtration rate; HR, hazard ratio; NGAL, neutrophil gelatinase-associated lipocalin; UAE, urinary albumin excretion.

Stratified analyses for the association between plasma NGAL and the risk of new-onset CKD were performed, which demonstrated consistently positive associations in almost all analyzed subgroups (Figure 3, Table S1). Stratification by the median of UAE, current smoking, and by eGFR (60–89 mL/min/1.73 m^2^ vs. eGFR ≥ 90 mL/min/1.73 m^2^) showed significant effect modification (*p*_interaction_ < 0.001, 0.034, and 0.001, respectively). The corresponding HRs were higher for participants with below-median UAE (<7.7 mg/24-h), for non-smokers, and for participants with a slightly reduced eGFR (60–89 mL/min/1.73 m^2^).

### 3.4. Sensitivity Analyses

The estimated association between plasma NGAL and the risk of new-onset CKD (composite outcome) when adjusted for potentially confounding variables (Model 4, Table 2) did not materially change when participants with UAE > 25 mg/24-h (instead of 30 mg/24-h) at baseline were excluded (HR per doubling 1.37 [95% CI: 1.08–1.74], *p* = 0.011). When participants with eGFR < 65 mL/min/1.73 m^2^ at baseline (instead of <60 mL/min/1.73 m^2^) were excluded, the association between plasma NGAL and the risk of new-onset CKD remained similar, albeit lost statistical significance (HR per doubling 1.21 [0.95–1.53], *p* = 0.121). When extreme outliers of plasma NGAL (<0.5th percentile and >99.5th percentile) were excluded, the association between plasma NGAL and the risk of new-onset CKD did not materially change (HR per doubling 1.41 [1.11–1.79], *p* = 0.005).

## 4. Discussion

The major finding of this study is that plasma NGAL concentrations are significantly associated with an increased risk of new-onset CKD in the general population within a follow-up of almost 10 years. This observation remained statistically significant when adjusting for known risk factors for CKD, including age, sex, history of CVD, diabetes, hypertension, and low-grade systemic inflammation (represented by hs-CRP). However, the association lost significance after adjustment for baseline eGFR, indicating that plasma NGAL is associated with new-onset CKD, albeit not independently of renal function. When considering individual determinants defining new-onset CKD (either eGFR < 60 mL/min/1.73 m^2^ or UAE > 30 mg/24-h) and their associations with plasma NGAL concentrations, we observed a considerably stronger association with eGFR < 60 mL/min/1.73 m^2^ as outcome, whereas associations with UAE > 30 mg/24-h were weaker and non-significant. Stratified analyses across relevant subgroups demonstrated almost consistently positive associations between plasma NGAL concentrations and risk of new-onset CKD. Significant interactions were found for the presence of hypertension, smoking, and eGFR (60–89 vs. ≥ 90 mL/min/1.73 m^2^) showing stronger associations in participants with below-median UAE, non-smokers, and participants with eGFR 60–89 mL/min/1.73 m^2^, respectively. Taken together, these results suggest that plasma NGAL concentrations are strongly associated with an increased risk of new-onset CKD, which is mainly driven by new-onset CKD as defined by renal function decline, and strongly dependent on baseline renal function. NGAL may potentially be of value as renal damage marker, aiding in risk stratification in preventive settings with the ultimate goal to combat future CKD burden.

NGAL is produced by both activated neutrophils and renal tubular cells, and may therefore be indicative, via different mechanisms, of low-grade systemic inflammation and tubular kidney injury, respectively. Under physiological conditions, circulating NGAL is filtered by the glomeruli and almost fully reabsorbed by proximal tubular epithelial cells [36]. Elevated levels of NGAL are regarded as indicator of ongoing renal damage, both in response to acute stressors (e.g., AKI) and in the context of (progression of) CKD [15,37]. Importantly, NGAL is abundantly released by tubular cells, resulting in increased circulating and urinary levels upon renal damage, often preceding a rise in serum creatinine, and thus providing opportunities for use as a biomarker of early kidney injury [10]. In addition, higher levels of plasma NGAL have also been shown to increase the risk of delayed graft function, graft failure and mortality in renal transplant recipients [9,38]. Urinary NGAL has also been associated with histological signs of renal damage in patients with CKD [13,39]. Although both urinary and circulating levels of NGAL have been extensively studied as (early) biomarker for AKI and CKD progression, its potential role as biomarker for pre-diagnostic CKD has only been investigated once in a small, matched case–control study [16]. To the best of our knowledge, the present prospective observational study with long-term follow-up is the largest to demonstrate that elevated plasma NGAL concentrations in subjects without CKD predispose to the development of future CKD. Although we were not able to pinpoint the exact origin of plasma NGAL levels (i.e., discriminating between different isoforms of the protein), the fact that NGAL may reflect both chronic low-grade systemic inflammation and renal tubular injury may render it a potentially useful clinical biomarker for new-onset CKD.

Interestingly, the association between plasma NGAL concentrations and the risk of new-onset CKD was primarily driven by new-onset CKD defined by an eGFR < 60 mL/min/1.73 m^2^. Furthermore, when we additionally adjusted our Cox proportional hazards regression models for eGFR at baseline, statistically significant relationships were abrogated. This is in line with other studies showing kidney function-dependency of NGAL concentrations across different patient populations [40,41,42]. The intimate relationship between NGAL levels and eGFR is sustained by studies that have shown that NGAL may represent a reliable biomarker of the severity of renal impairment, i.e., could represent a surrogate marker of kidney damage [11,43,44]. Previously, a theory referred to as the “forest fire” hypothesis has been proposed to explain the relationship between NGAL and eGFR [14]. This theory states that higher levels of NGAL in patients with CKD (the “forest fire”) is the mere consequence of a sustained production by inflamed but viable renal tubular cells, whereas the rise in serum creatinine levels and decline in eGFR are rather passive consequences of a general loss of functional cells or nephrons. As such, NGAL has been suggested to function as a “real-time indicator” of the extent of active renal damage within the context of the overall condition of CKD [10]. In the present study, we observed a significant association between higher levels of plasma NGAL and reduced eGFR at baseline and, prospectively, we observed higher plasma NGAL levels in individuals having a higher risk of developing new-onset CKD (as (partially) defined by eGFR). As such, it was not possible to discriminate between NGAL as biomarker of both impaired renal function and more rapid development of CKD, and the possibility that the association between NGAL and new-onset CKD was merely confounded by baseline eGFR. Considering that our study was of associative nature, future studies are warranted to fill this knowledge gap and disentangle the exact sequence of events in the association between NGAL, renal function (eGFR) and new-onset CKD.

Previous studies have shown a clear relationship between circulating NGAL levels and hs-CRP, obviously because of neutrophilic activation in circumstances of low-grade systemic inflammation [42]. In the current study, the association between plasma NGAL and the risk of new-onset CKD was independent of hs-CRP levels, a finding that is in line with a recent study from our center of renal transplant recipients [9]. This may suggest that, although NGAL and inflammation are intricately related, the presence of increased NGAL or hs-CRP may reflect different types of inflammation. Whereas hs-CRP can be regarded as a rather generic marker of systemic inflammation, NGAL may be a more specific marker of intrarenal inflammation, especially given its strong association with estimated glomerular filtration rate. Although the assay used to measure NGAL could not discriminate between neutrophil-derived and renal tubular cell-derived NGAL, it is abundantly expressed by tubular renal cells and especially upon tubulointerstitial injury [13,39].

Stratified analyses showed that plasma NGAL concentrations and the risk of new-onset CKD remained consistently positively associated with each other across all subgroups, except for smoking participants. Of note, associations between plasma NGAL and the risk of new-onset CKD were stronger in participants who smoked, in participants with below-median UAE, and in participants with a slightly reduced eGFR (60–89 mL/min/1.73 m^2^), which all demonstrated significant interactions. Smoking has been variably associated with plasma NGAL levels in different clinical contexts [9,38,45], and in this study the frequency of current smokers was significantly higher in the highest tertile of NGAL concentrations. The weaker associations between plasma NGAL and risk of new-onset CKD in smoking participants and those with relatively higher baseline UAE may indicate that elevated NGAL levels due to such factors decrease their variation, and, thus, induce a loss of predictive performance regarding new-onset CKD. Since NGAL may be a marker of both activated neutrophils and ongoing renal damage, these findings suggest that this marker is of most clinical significance in participants without too many risk factors for developing CKD, but with a slightly reduced renal function. The latter observation also sustains the observation that statistical significance of the associations between plasma NGAL and new-onset CKD was lost after additional adjustment for baseline eGFR, indicating the strong risk of developing CKD associated with baseline eGFR. Findings from these stratified analyses should however be interpreted with caution, since sample sizes were smaller in subgroups, and sometimes also rather imbalanced, which may introduce bias.

A potentially relevant strength of this study is that it clearly demonstrated that plasma NGAL might be an alternative biomarker of renal function but might not be suitable as surrogate index of renal function. Another strength relates to the large number of study participants and cases of new-onset CKD in this extensively characterized dataset, allowing to adjust for many potential confounding variables in a prospective setting. However, some limitations of the study also warrant recognition. First, this was a single-center study without much variation in ethnicity or geographic location, as most participants were Caucasians and lived in the northern part of the Netherlands, limiting the generalizability of our results. Second, we were forced to exclude study participants of whom no sufficient plasma samples were available anymore to determine plasma NGAL. Third, the NGAL immunoassay used in this study did not allow us to make a distinction between monomeric and dimeric forms of NGAL, which could make a difference from a pathophysiological perspective [46]. Finally, since this study was of a longitudinal observational nature, we were not able to prove causality in our findings, but instead could only document associations. Considering the well-characterized phenotypes of this study cohort, we were notably able to reliably adjust for the relevant confounding factors, albeit residual confounding cannot be fully excluded.

## 5. Conclusions

In conclusion, our results highlight a significant association between plasma NGAL concentrations and an increased risk of new-onset CKD in the general population. In this cohort of population-based individuals, plasma NGAL seems to have no added value beyond the assessment of renal function (eGFR) to predict the future occurrence of CKD. This might, however, be different in patients with impaired renal function or specific kidney diseases. The present data support the performance of such studies. Therefore, future efforts are needed to examine the potential value of NGAL in different clinical populations and to compare the potential clinical utility of urinary NGAL versus plasma NGAL concentrations in relation to the risk of new-onset CKD.

## Figures and Tables

**Table 1:** Baseline demographic, clinical and biochemical characteristics and outcomes of the study population, stratified by tertiles of plasma NGAL concentrations.

	Total	T1	T2	T3	*p*-Value
		♂: <89.9 μg/L ♀: <85.7 μg/L	♂: 89.9–113.9 μg/L ♀: 85.7–111.1μg/L	♂: >113.9 μg/L ♀: >111.1 μg/L	
Plasma NGAL (μg/L)	104.0 (34.7)	71.0 (13.6)	99.6 (7.2)	141.4 (29.4)	<0.001
**Demographics**					
Age (years)	50.0 [42.2–58.8]	50.5 [42.1–58.3]	50.3 [42.6–59.0]	49.2 [42.0–59.2]	0.653
Female, *n* (%)	2513 (53.9)	896 (57.7)	817 (52.6)	800 (51.5)	0.001
Race, n (%)					<0.001
*White, n (%)*	4439 (96.0)	1438 (93.1)	1487 (96.6)	1514 (98.2)	
*Black, n (%)*	42 (0.9)	24 (1.6)	9 (0.6)	9 (0.6)	
*Asian, n (%)*	94 (2.0)	61 (3.9)	23 (1.5)	10 (0.6)	
*Other, n (%)*	51 (1.1)	22 (1.4)	20 (1.3)	9 (0.6)	
**Anthropometrics**					
BMI (kg/m^2^)	25.7 [23.4–28.4]	25.8 [23.3–28.5]	25.7 [23.4–28.4]	25.7 [23.4–28.4]	0.992
Waist circumference (cm)	90 [81–98]	89 [80–98]	90 [82–98]	90 [82–99]	0.003
**Cardiovascular risk factors**					
SBP (mmHg)	120 [111–133]	121 [111–133]	121 [111–134]	120 [110–132]	0.339
DBP (mmHg)	72 [66–78]	72 [66–78]	72 [66–78]	71 [66–77]	0.129
Heart rate (bpm)	68 [62–74]	67 [61–73]	68 [62–74]	68 [62–75]	<0.001
Smoking					<0.001
*Never, n (%)*	1449 (31.5)	552 (35.9)	499 (32.6)	398 (25.9)	
*Current, n (%)*	1283 (27.9)	329 (21.4)	399 (26.1)	555 (36.2)	
*Former, n (%)*	1870 (40.6)	656 (42.7)	632 (41.3)	582 (37.9)	
History of CVD, *n* (%)	123 (97.1)	37 (2.4)	41 (2.6)	45 (2.9)	0.672
Diabetes, *n* (%)	68 (1.5)	24 (1.6)	21 (1.4)	23 (1.5)	0.900
Hypertension, *n* (%)	1166 (25.0)	398 (25.6)	390 (25.1)	378 (24.3)	0.705
**Medication**					
Antihypertensive drugs, *n* (%)	624 (13.8)	220 (14.5)	201 (13.4)	203 (13.4)	0.361
Lipid-lowering drugs, *n* (%)	272 (5.8)	95 (6.1)	79 (5.1)	98 (6.3)	0.296
Glucose-lowering drugs, *n* (%)	39 (1.0)	15 (1.1)	12 (0.9)	12 (0.9)	0.774
**Laboratory measurements**					
Total cholesterol (mmol/L)	5.36 [4.72–6.09]	5.43 [4.79–6.19]	5.31 [4.68–6.05]	5.33 [4.69–6.06]	0.002
hs-CRP (mg/L)	1.16 [0.56–2.65]	0.91 [0.42–1.84]	1.09 [0.53–2.40]	1.77 [0.80–3.85]	<0.001
eGFR (mL/min/1.73 m^2^)	96.1 [85.3–105.8]	99.3 [88.7–108.6]	96.0 [85.2–105.7]	93.0 [80.8–102.3]	<0.001
UAE (mg/L)	7.7 [5.8–11.2]	7.8 [5.9–11.0]	7.7 [5.8–11.4]	7.5 [5.6–11.2]	0.409
Serum creatinine (μmol/l)	81.1 [72.9–90.4]	79.1 [70.8–88.3]	81.1 [73.9–90.4]	84.2 [74.9–93.4]	<0.001
Urine creatinine (mmol/24-h)	11.9 [9.9–14.5]	11.7 [9.8–14.7]	12.0 [10.1–14.5]	11.9 [9.9–14.4]	0.317
**Study outcomes**					
CKD (eGFR < 60 mL/min/1.73 m^2^), *n* (%)	151 (3.3)	33 (2.1)	51 (3.3)	67 (4.4)	0.002
CKD (UAE >30 mg/24-h), *n* (%)	349 (7.6)	109 (7.1)	115 (7.4)	125 (8.2)	0.483
CKD (combined), *n* (%)	467 (10.1)	132 (8.6)	161 (10.4)	174 (11.4)	0.029

Abbreviations: BMI, body-mass index; CKD, chronic kidney disease; CVD, cardiovascular disease; DBP, diastolic blood pressure; eGFR, estimated glomerular filtration rate; hs-CRP, high-sensitive C-reactive protein; SBP, systolic blood pressure; UAE, urinary albumin excretion.

**Table 2:** Cox proportional hazards regression analyses for associations between plasma NGAL and the risk of incident CKD, either as composite outcome or based on its individual determinants (eGFR and UAE).

	HR per Doubling	T1	T2	T3
		<87.6 μg/L	87.6–112.6 μg/L	>112.6 μg/L
**A. CKD (composite outcome) (*n* = 467)**				
Model 1	1.35 [1.11–1.63], ***p* = 0.002**	1.00 (reference)	1.24 [0.99–1.57], *p* = 0.063	1.44 [1.15–1.81], ***p* = 0.002**
Model 2	1.36 [1.12–1.65], ***p* = 0.002**	1.00 (reference)	1.18 [0.94–1.49], *p* = 0.150	1.46 [1.16–1.83], ***p* = 0.001**
Model 3	1.36 [1.12–1.66], ***p* = 0.002**	1.00 (reference)	1.22 [0.96–1.53], *p* = 0.099	1.50 [1.19–1.88], ***p* < 0.001**
Model 4	1.37 [1.09–1.73], ***p* = 0.007**	1.00 (reference)	1.19 [0.92–1.55], *p* = 0.191	1.51 [1.16–1.96], ***p* = 0.002**
Model 5	1.09 [0.86–1.37], *p* = 0.490	1.00 (reference)	1.05 [0.80–1.37], *p* = 0.726	1.16 [0.89–1.53], *p* = 0.274
**B. CKD (eGFR < 60 mL/min/1.73 m^2^) (*n* = 151)**				
Model 1	2.07 [1.47–2.91], ***p* < 0.001**	1.00 (reference)	1.56 [1.01–2.42], ***p* = 0.046**	2.20 [1.45–3.34], ***p* < 0.001**
Model 2	2.42 [1.70–3.46], ***p* < 0.001**	1.00 (reference)	1.50 [0.96–2.32], *p* = 0.073	2.48 [1.63–3.78], ***p* < 0.001**
Model 3	2.35 [1.66–3.34], ***p* < 0.001**	1.00 (reference)	1.54 [0.99–2.39], *p* = 0.056	2.49 [1.63–3.80], ***p* < 0.001**
Model 4	2.54 [1.69–3.80], ***p* < 0.001**	1.00 (reference)	1.54 [0.95–2.50], *p* = 0.079	2.55 [1.60–4.06], ***p* < 0.001**
Model 5	1.05 [0.69–1.59], *p* = 0.828	1.00 (reference)	0.89 [0.54–1.45], *p* = 0.633	0.98 [0.61–1.58], *p* = 0.929
**C. CKD (UAE > 30 mg/24-h) (*n* = 349)**				
Model 1	1.21 [0.98–1.50], *p* = 0.080	1.00 (reference)	1.08 [0.83–1.40], *p* = 0.573	1.27 [0.98–1.64], *p* = 0.068
Model 2	1.15 [0.93–1.43], *p* = 0.210	1.00 (reference)	1.02 [0.79–1.33], *p* = 0.814	1.22 [0.94–1.57], *p* = 0.132
Model 3	1.15 [0.92–1.43], *p* = 0.214	1.00 (reference)	1.05 [0.81–1.37], *p* = 0.710	1.23 [0.95–1.59], *p* = 0.114
Model 4	1.07 [0.83–1.37], *p* = 0.609	1.00 (reference)	1.02 [0.75–1.39], *p* = 0.892	1.18 [0.87–1.60], *p* = 0.296
Model 5	1.07 [0.82–1.40], *p* = 0.604	1.00 (reference)	1.03 [0.76–1.40], *p* = 0.864	1.19 [0.87–1.64], *p* = 0.279

Model 1, crude model. Model 2, model 1 with adjustment for age and sex. Model 3, model 2 with adjustment for history of cardiovascular disease, history of diabetes, and the presence of hypertension. Model 4, model 3 with adjustment for hs-CRP. Model 5, model 4 with additional adjustment for baseline eGFR. Bold *p*-values indicate statistical significance. Abbreviations: HR, hazard ratio; CKD, chronic kidney disease; eGFR, estimated glomerular filtration rate; UAE, urinary albumin excretion.

## Data Availability

The datasets generated for this study are available on reasonable request to the corresponding author.

## Supplementary Materials

The following supporting information can be downloaded at: https://www.mdpi.com/article/10.3390/biom13020338/s1, Table S1: Stratified analyses for the association between plasma NGAL concentrations and the risk of incident CKD across various subgroups.

## References

[1] Kidney disease: Improving global outcomes (KDIGO) CKD work group (2013). KDIGO 2012 clinical practice guideline for the evaluation and management of chronic kidney disease. Kidney Int. Suppl..

[2] Shlipak M.G., Day E.C. (2013). Biomarkers for incident CKD: A new framework for interpreting the literature. Nat. Rev. Nephrol..

[3] Gansevoort R.T., de Jong P.E. (2009). The case for using albuminuria in staging chronic kidney disease. J. Am. Soc. Nephrol..

[4] Cowland J.B., Borregaard N. (1997). Molecular characterization and pattern of tissue expression of the gene for neutrophil gelatinase-associated lipocalin from humans. Genomics.

[5] Banai A., Rozenfeld K.-L., Levit D., Merdler I., Loewenstein I., Banai S., Shacham Y. (2020). Neutrophil gelatinase-associated lipocalin (NGAL) for the prediction of acute kidney injury in chronic kidney disease patients treated with primary percutaneous coronary intervention. Int. J. Cardiol. Heart Vasc..

[6] Lumlertgul N., Amprai M., Tachaboon S., Dinhuzen J., Peerapornratana S., Kerr S.J., Srisawat N. (2020). Urine Neutrophil Gelatinase-associated Lipocalin (NGAL) for Prediction of Persistent AKI and Major Adverse Kidney Events. Sci. Rep..

[7] Cruz D.N., De Cal M., Garzotto F., Perazella M.A., Lentini P., Corradi V., Piccinni P., Ronco C. (2010). Plasma neutrophil gelatinase-associated lipocalin is an early biomarker for acute kidney injury in an adult ICU population. Intensiv. Care Med..

[8] Mishra J., Dent C., Tarabishi R., Mitsnefes M.M., Ma Q., Kelly C., Ruff S.M., Zahedi K., Shao M., Bean J. (2005). Neutrophil gelatinase-associated lipocalin (NGAL) as a biomarker for acute renal injury after cardiac surgery. Lancet.

[9] Kremer D., Post A., Gomes-Neto A.W., Groothof D., Kunutsor S.K., Nilsen T., Hidden C., Sundrehagen E., Eisenga M.F., Navis G. (2021). Plasma neutrophil gelatinase-associated lipocalin and kidney graft outcome. Clin. Kidney J..

[10] Bolignano D., Lacquaniti A., Coppolino G., Donato V., Campo S., Fazio M.R., Nicocia G., Buemi M. (2009). Neutrophil gelatinase-associated lipocalin (NGAL) and progression of chronic kidney disease. Clin. J. Am. Soc. Nephrol..

[11] Bolignano D., Coppolino G., Campo S., Aloisi C., Nicocia G., Frisina N., Buemi M. (2008). Urinary neutrophil gelatinase-associated lipocalin (NGAL) is associated with severity of renal disease in proteinuric patients. Nephrol. Dial. Transplant..

[12] Mitsnefes M.M., Kathman T.S., Mishra J., Kartal J., Khoury P.R., Nickolas T.L., Barasch J., Devarajan P. (2007). Serum neutrophil gelatinase-associated lipocalin as a marker of renal function in children with chronic kidney disease. Pediatr. Nephrol..

[13] Ding H., He Y., Li K., Yang J., Li X., Lu R., Gao W. (2017). Urinary neutrophil gelatinase-associated lipocalin (NGAL) is an early biomarker for renal tubulointerstitial injury in IgA nephropathy. Clin. Immunol..

[14] Mori K., Nakao K. (2007). Neutrophil gelatinase-associated lipocalin as the real-time indicator of active kidney damage. Kidney Int..

[15] Bolignano D., Donato V., Coppolino G., Campo S., Buemi A., Lacquaniti A., Buemi M. (2008). Neutrophil gelatinase-associated lipocalin (NGAL) as a marker of kidney damage. Am. J. Kidney Dis..

[16] Bhavsar N.A., Köttgen A., Coresh J., Astor B.C. (2012). Neutrophil gelatinase-associated lipocalin (NGAL) and kidney injury molecule 1 (KIM-1) as predictors of incident CKD stage 3: The Atherosclerosis Risk in Communities (ARIC) Study. Am. J. Kidney Dis..

[17] Hillege H.L., Janssen W.M.T., Bak A.A.A., Diercks G.F.H., Grobbee D.E., Crijns H.J.G.M., Van Gilst W.H., De Zeeuw D., De Jong P.E. (2001). Microalbuminuria is common, also in a nondiabetic, nonhypertensive population, and an independent indicator of cardiovascular risk factors and cardiovascular morbidity. J. Intern. Med..

[18] von Elm E., Altman D.G., Egger M., Pocock S.J., Gøtzsche P.C., Vandenbroucke J.P. (2007). STROBE Initiative. The Strengthening the Reporting of Observational Studies in Epidemiology (STROBE) statement: Guidelines for reporting observational studies. Ann Intern. Med..

[19] Post A., Kremer D., Swarte J.C., Sokooti S., Vogelpohl F.A., Groothof D., Kema I., Garcia E., Connelly M.A., Wallimann T. (2021). Plasma creatine concentration is associated with incident hypertension in a cohort enriched for the presence of high urinary albumin concentration: The Prevention of Renal and Vascular Endstage Disease study. J. Hypertens..

[20] Bourgonje A.R., Bourgonje M.F., Post A., Gemert S.L.B.-V., Kieneker L.M., Bulthuis M.L., Gordijn S.J., Gansevoort R.T., Bakker S.J.L., Mulder D.J. (2022). Systemic oxidative stress associates with new-onset hypertension in the general population. Free. Radic. Biol. Med..

[21] Kappelle P.J.W.H., Gansevoort R.T., Hillege J.L., Wolffenbuttel B.H.R., Dullaart R.P.F. (2011). PREVEND study group. Apolipoprotein B/A-I and total cholesterol/high-density lipoprotein cholesterol ratios both predict cardiovascular events in the general population independently of nonlipid risk factors, albuminuria and C-reactive protein. J. Intern. Med..

[22] Borggreve S.E., Hillege H.L., Wolffenbuttel B.H.R., De Jong P.E., Bakker S.J.L., Van Der Steege G., Van Tol A., Dullaart R.P.F. (2005). The effect of cholesteryl ester transfer protein -629C->A promoter polymorphism on high-density lipoprotein cholesterol is dependent on serum triglycerides. J. Clin. Endocrinol. Metab..

[23] Grubb A., Blirup-Jensen S., Lindström V., Schmidt C., Althaus H., Zegers I. (2010). IFCC Working Group on Standardisation of Cystatin C (WG-SCC). First certified reference material for cystatin C in human serum ERM-DA471/IFCC. Clin. Chem. Lab. Med..

[24] Blirup-Jensen S., Johnson A.M., Larsen M. (2008). IFCC Committee on Plasma Proteins. Protein standardization V: Value transfer. A practical protocol for the assignment of serum protein values from a Reference Material to a Target Material. Clin. Chem. Lab. Med..

[25] Salvagno G.L., Ferrari A., Gelati M., Brocco G., Lippi G. (2017). Analytical validation of Gentian NGAL particle-enhanced enhanced turbidimetric immunoassay (PETIA). Pract. Lab. Med..

[26] Inker L.A., Schmid C.H., Tighiouart H., Eckfeldt J.H., Feldman H.I., Greene T., Kusek J.W., Manzi J., Van Lente F., Zhang Y.L. (2012). Estimating glomerular filtration rate from serum creatinine and cystatin C. N. Engl. J. Med..

[27] Abbasi A., Corpeleijn E., Gansevoort R.T., Gans R., Hillege H.L., Stolk R., Navis G., Bakker S.J.L., Dullaart R.P.F. (2013). Role of HDL cholesterol and estimates of HDL particle composition in future development of type 2 diabetes in the general population: The PREVEND study. J. Clin. Endocrinol. Metab..

[28] Gemert S.L.B.-V., Heuvel E.V.D. (2013). Exploring causal hypotheses: Breaking with long-standing research traditions. Dev. Med. Child Neurol..

[29] Leopold J.A. (2015). The Central Role of Neutrophil Gelatinase–Associated Lipocalin in Cardiovascular Fibrosis. Hypertension.

[30] Lindberg S., Jensen J.S., Hoffmann S., Iversen A.Z., Pedersen S.H., Biering-Sørensen T., Galatius S., Flyvbjerg A., Mogelvang R., Magnusson N.E. (2016). Plasma Neutrophil Gelatinase-Associated Lipocalin Reflects Both Inflammation and Kidney Function in Patients with Myocardial Infarction. Cardiorenal Med..

[31] Mosialou I., Shikhel S., Luo N., Petropoulou P.I., Panitsas K., Bisikirska B., Rothman N.J., Tenta R., Cariou B., Wargny M. (2020). Lipocalin-2 counteracts metabolic dysregulation in obesity and diabetes. J. Exp. Med..

[32] Moschen A.R., Adolph T.E., Gerner R.R., Wieser V., Tilg H. (2017). Lipocalin-2: A Master Mediator of Intestinal and Metabolic Inflammation. Trends Endocrinol. Metab..

[33] Iqbal N., Choudhary R., Chan J., Wentworth B., Higginbotham E., Maisel A.S. (2013). Neutrophil gelatinase-associated lipocalin as diagnostic and prognostic tool for cardiovascular disease and heart failure. Expert Opin. Med Diagn..

[34] Castillo-Rodriguez E., Fernandez-Prado R., Martin-Cleary C., Pizarro-Sánchez M.S., Sanchez-Niño M.D., Sanz A.B., Fernandez-Fernandez B., Ortiz A. (2017). Kidney Injury Marker 1 and Neutrophil Gelatinase-Associated Lipocalin in Chronic Kidney Disease. Nephron.

[35] Chong J.J.H., Prince R.L., Thompson P.L., Thavapalachandran S., Ooi E., Devine A., Lim E.E.M., Byrnes E., Wong G., Lim W.H. (2019). Association Between Plasma Neutrophil Gelatinase-Associated Lipocalin and Cardiac Disease Hospitalizations and Deaths in Older Women. J. Am. Heart Assoc..

[36] Kuwabara T., Mori K., Mukoyama M., Kasahara M., Yokoi H., Saito Y., Yoshioka T., Ogawa Y., Imamaki H., Kusakabe T. (2009). Urinary neutrophil gelatinase-associated lipocalin levels reflect damage to glomeruli, proximal tubules, and distal nephrons. Kidney Int..

[37] Haase M., Bellomo R., Devarajan P., Schlattmann P., Haase-Fielitz A. (2009). NGAL Meta-analysis Investigator Group. Accuracy of neutrophil gelatinase-associated lipocalin (NGAL) in diagnosis and prognosis in acute kidney injury: A systematic review and meta-analysis. Am. J. Kidney Dis..

[38] Haase-Fielitz A., Haase M. (2014). Neutrophil gelatinase-associated lipocalin as a biomarker of acute kidney injury: A critical evaluation of current status. Ann. Clin. Biochem..

[39] Yavas H., Sahin O.Z., Ersoy R., Taşlı F., Genek D.G., Uzum A., Cirit M. (2013). Prognostic value of NGAL staining in patients with IgA nephropathy. Ren. Fail..

[40] Damman K., Van Veldhuisen D.J., Navis G., Voors A.A., Hillege H.L. (2008). Urinary neutrophil gelatinase associated lipocalin (NGAL), a marker of tubular damage, is increased in patients with chronic heart failure. Eur. J. Heart Fail..

[41] Kunutsor S.K., Flores-Guerrero J.L., Kieneker L.M., Nilsen T., Hidden C., Sundrehagen E., Seidu S., Dullaart R.P., Bakker S.J. (2018). Plasma neutrophil gelatinase-associated lipocalin and risk of cardiovascular disease: Findings from the PREVEND prospective cohort study. Clin. Chim. Acta.

[42] Daniels L.B., Barrett-Connor E., Clopton P., Laughlin G.A., Ix J.H., Maisel A.S. (2012). Plasma neutrophil gelatinase-associated lipocalin is independently associated with cardiovascular disease and mortality in community-dwelling older adults: The Rancho Bernardo Study. J. Am. Coll. Cardiol..

[43] Bolignano D., Coppolino G., Campo S., Aloisi C., Nicocia G., Frisina N., Buemi M. (2007). Neutrophil gelatinase-associated lipocalin in patients with autosomal-dominant polycystic kidney disease. Am. J. Nephrol..

[44] Bolignano D., Lacquaniti A., Coppolino G., Campo S., Arena A., Buemi M. (2008). Neutrophil gelatinase-associated lipocalin reflects the severity of renal impairment in subjects affected by chronic kidney disease. Kidney Blood Press. Res..

[45] Eagan T.M., Damås J.K., Ueland T., Voll-Aanerud M., Mollnes T.E., Hardie J.A., Bakke P.S., Aukrust P. (2010). Neutrophil gelatinase-associated lipocalin: A biomarker in COPD. Chest.

[46] Cai L., Rubin J., Han W., Venge P., Xu S. (2010). The origin of multiple molecular forms in urine of HNL/NGAL. Clin. J. Am. Soc. Nephrol..

