# Supplementary material for: Plasma Neutrophil Gelatinase-Associated Lipocalin Associates with New-Onset Chronic Kidney Disease in the General Population
Source: Biomolecules. 2023 Feb 9;13(2):338. doi: 10.3390/biom13020338 (PMC9953575; doi:10.3390/biom13020338)
Supplement: Supplementary file 1 [file biomolecules-13-00338-s001.zip › Table S1.pdf]

Table S1. Stratified analyses for the association between plasma NGAL concentrations and the risk of incident CKD across various subgroups.

**Variable**	**Total (*n*)**	**New-onset CKD (*n*)**	**HR (95% CI)**	***P*-value for interaction**
**Overall**	4,660	467	1.37 [1.09-1.73]	
**Gender**				
Male	2,147	266	1.36 [0.98-1.88]	0.919
Female	2,513	201	1.31 [0.95-1.81]	
**BMI**				
< 25 kg/m^2	1,976	163	1.57 [1.06-2.34]	0.671
≥ 25 kg/m^2	2,684	304	1.29 [0.97-1.72]	
**Hypertension**				
No	3,492	263	1.20 [0.89-1.62]	0.113
Yes	1,166	203	1.68 [1.19-2.36]	
**History of CVD**				
No	4,537	445	1.37 [1.09-1.73]	0.805

Yes	123	22	1.12 [0.34-3.66]	
**UAE**				
< 7.7 mg/24-h	2,330	96	1.69 [1.04-2.73]	**<0.001**
> 7.7 mg/24-h	2,330	371	1.36 [1.04-1.77]	
**eGFR**				
60-89 mL/min/1.73m^2	1,614	264	1.38 [1.01-1.89]	**<0.001**
≥ 90 mL/min/1.73m^2	2,998	203	1.09 [0.78-1.52]	
**Current smoking**				
No	3,319	329	1.67 [1.25-2.22]	**0.034**
Yes	1,283	130	0.78 [0.52-1.17]	
**Total cholesterol**				
< 5.36 mmol/L	2,330	210	1.52 [1.07-2.15]	0.878
> 5.36 mmol/L	2,330	256	1.22 [0.90-1.66]	

Abbreviations: CKD, chronic kidney disease; CI, confidence interval; HR, hazard ratio; eGFR, estimated glomerular filtration rate; UAE, urinary albumin excretion; BMI, body-mass index; CHF, chronic heart failure.
